# Treatment Outcome of a Combined Dose-Escalated Treatment Regime With Helical TomoTherapy® and Active Raster-Scanning Carbon Ion Boost for Adenocarcinomas of the Head and Neck

**DOI:** 10.3389/fonc.2019.00755

**Published:** 2019-08-13

**Authors:** Sati Akbaba, Andreas Mock, Juliane Hoerner-Rieber, Thomas Held, Sonja Katayama, Tobias Forster, Christian Freudlsperger, Stefan Rieken, Klaus Herfarth, Peter Plinkert, Juergen Debus, Sebastian Adeberg

**Affiliations:** ^1^Department of Radiation Oncology, University Hospital Heidelberg, Heidelberg, Germany; ^2^Department of Radiation Oncology, Heidelberg Institute of Radiation Oncology, Heidelberg, Germany; ^3^National Center for Tumor Diseases, Heidelberg, Germany; ^4^Heidelberg Ion-Beam Therapy Center, Heidelberg, Germany; ^5^Department of Medical Oncology, National Center for Tumor Diseases Heidelberg, Heidelberg, Germany; ^6^Department of Translational Medical Oncology, National Center for Tumor Diseases Heidelberg, German Cancer Research Center, Heidelberg, Germany; ^7^Clinical Cooperation Unit Radiation Oncology, German Cancer Research Center, Heidelberg, Germany; ^8^Department of Oral and Maxillofacial Surgery, Heidelberg University Hospital, Heidelberg, Germany; ^9^Department of Otorhinolaryngology, Head and Neck Surgery, University Hospital Heidelberg, Heidelberg, Germany

**Keywords:** salivary gland adenocarcinoma, salivary duct adenocarcinoma, intestinal-type adenocarcinoma, carbon ion radiotherapy, tomotherapy, local control, side effects

## Abstract

**Introduction:** Data regarding treatment and survival outcome of patients with adenocarcinoma of the head and neck are limited to case reports and case series. As a consequence of lacking evidence, treatment guidelines do not exist. We aimed to analyze the effect of a bimodal irradiation regime with intensity modulated radiotherapy (IMRT) and carbon ion boost on local control (LC) and survival in adenocarcinoma patients for a large patient collective.

**Materials and Methods:** Patient records of eighty consecutive patients treated between 2009 and 2018 were analyzed retrospectively and Kaplan-Meier estimates for LC, overall survival (OS) and progression-free survival (PFS) were compared among patients with salivary gland adenocarcinoma (SGAC), salivary duct adenocarcinoma (SDAC), and intestinal-type adenocarcinoma (ITAC) according to the World Health Organization (WHO). Prognostic factors were identified using the log-rank test and cox-regression modeling. Toxicity was assessed according to the Common Terminology Criteria for Adverse Events (CTCAE).

**Results:** Median follow-up was 41 months. The 3-year and estimated 5-year Kaplan-Meier rates for all patients were 83 and 75% for LC, 74 and 50% for OS and 60 and 53% for PFS, respectively. While bimodal RT for ITAC resulted in a significantly decreased 3-year LC rate of 50 vs. 93% for each SGAC and SDAC (*p* < 0.01), no statistical significant survival differences could be identified across the three groups regarding OS (*p* = 0.08) and PFS (*p* = 0.063). 3-year OS was 88% for SGAC, 78% for SDAC and 67% for ITAC and 3-year PFS was 72% for SGAC, 53% for SDAC and 44% for ITAC, respectively. Nevertheless, in subgroup analysis, OS for ITAC was significantly worse compared to SGAC (*p* = 0.024). In multivariate analysis, bilateral tumor side (vs. unilateral) solely could be identified as independent negative prognostic factor for LC (*p* < 0.01). Treatment was well-tolerated with 21% acute (*n* = 17) and 25% (*n* = 20) late grade ≥3 toxicities.

**Conclusion:** Radiotherapy including active raster-scanning carbon ion boost for relatively radio resistant adenocarcinomas of the head and neck resulted in favorable survival outcome for salivary gland and salivary duct adenocarcinomas with moderate toxicity. However, local control and prognosis for bilateral intestinal-type adenocarcinomas (ITAC) seem to remain low even after dose-escalation.

## Introduction

Adenocarcinomas of the head and neck represent a very heterogeneous group of tumors consisting of various sites of origin and histology (1, 2). In general, the 2017 World Health Organization (WHO) system classifies adenocarcinomas into major salivary gland adenocarcinomas (SGAC), including high-grade salivary duct adenocarcinomas (SDAC), minor SGACs as well as intestinal-type adenocarcinomas (ITAC) and non-intestinal-type adenocarcinomas (non-ITAC) of the nasal cavity and paranasal sinuses (2, 3).

SGACs are the third common salivary gland carcinoma (SGC) with an incidence of nearly 15% of all malignant SGCs in the head and neck. In the majority of cases, they arise from major salivary glands while only 25% are seated in the minor salivary glands of the paranasal sinuses, palate, oral cavity, larynx, or pharynx (1, 4, 5). Overall, malignant SGCs of the head and neck have a low incidence of 0.05–2 per 100.000 with an increasing incidence rate over the last decades (3, 6). Nutrition, radiation, immunosuppression, Epstein Barr virus (EBV), human immunodeficiency virus (HIV), human papillomavirus (HPV), and a malignant transformation of a pre-existing benign tumor are mostly discussed as etiological factors (7–9). SDACs arise from ductal epithelial cells of the salivary glands and account for 1–3% of all malignant SGCs (10). They are known as relatively aggressive tumors with a high lymphogenic and hematogenous metastasis rate and a poor prognosis (11, 12). Etiology of SDACs still remains unclear but a substantial proportion can be lead back to carcinomas ex pleomorphic adenoma. In contrast to SGAC and SDAC, ITACs do not arise from the salivary glands but originate in 98% of the cases from epithelial cells in the nasal cavity or paranasal sinuses. They are estimated to have an incidence of 0.5–1.5 per 100.000 accounting for nearly 3% of all malignancies in the head and neck (13). ITACs are strongly associated with prolonged exposure with wood and leather dusts mostly related to profession.

Due to the rareness of adenocarcinoma of the head and neck, treatment guidelines are lacking. Complete surgical resection is considered the mainstay treatment. Radiotherapy (RT) is generally required in case of inoperable tumors or postoperatively for advanced tumors with incomplete resection margins and other factors, i.e., T3/4, N+, PNI (perineural invasion) (13, 14). Adenocarcinomas are known for their relative radio resistance, that high RT doses are required. Dose delivery to the tumor or tumor bed is strongly limited by surrounding organs at risk, especially in the head, and neck. Therefore, we have treated patients with adenocarcinoma of the head and neck with a combined radiation treatment including intensity modulated RT and carbon ion boost for dose-escalation since 2009 in our institution, as carbon ions are known for their higher biological effectiveness and more conformal dose delivery compared to photons (15). In the current study, we purposed to present treatment results of this special combination regime of RT with or without surgery for the most common three groups of adenocarcinoma of the head and neck; SGAC, SDAC and ITAC.

## Materials and Methods

### Evaluation

Patient records of eighty consecutive patients with adenocarcinoma of the head and neck who received bimodal RT including intensity modulated RT (IMRT) at the Department of Radiation Oncology, University Hospital Heidelberg and carbon ion boost at the Heidelberg Ion-Beam Therapy Center (HIT) between 2009 and 2018 were analyzed retrospectively. Patients received either postoperative or definite RT in a primary or secondary setting.

Patients were followed-up every 3 months during the first 2 years after RT, every half year during the third year after RT and then, once a year with clinical examination by an otorhinolaryngologist as well as contrast-enhanced magnetic resonance imaging (MRI). A computed tomography (CT) was requested yearly to exclude distant failure. Toxicity was assessed according to Common Terminology Criteria for Adverse Events (CTCAE) version 5 and tumor response [stable disease (SD), complete remission (CR), partial remission (PR)] according to Response Evaluation Criteria in Solid Tumors (RECIST). Acute toxicity was defined as toxicity, which occurred during RT and 6 weeks after RT and late toxicity as toxicity, which was reported ≥ 3 months after RT.

Statistical tests were conducted with SPSS Statistics version 24 (IBM, Armonk, New York, USA) and R version 3.4.2 (www.r-project.org). A *p*-value of <0.05 was considered as statistically significant. Local control (LC) was assessed from time of RT up to local progression. Overall survival (OS) and progression-free survival (PFS) were calculated from the first diagnosis to the last follow-up or time of event (death for OS; death/local/regional/distant progression for PFS). In addition, regional control from time of RT to time of regional relapse into nodes in the neck (RC) and distant control from time of RT to time of distant relapse (DC) were assessed. Kaplan-Meier estimates of potential prognostic factors were compared using the log-rank test for univariate and the cox-regression model for multivariate analysis.

### Patient and Tumor Characteristics

The patient and tumor characteristics of the patient cohort are shown in Table 1. According to WHO, patients were divided into three histological groups of adenocarcinoma of the head and neck for analysis; SGAC, SDAC, and ITAC (for further histological subtyping in the SGAC group, please see Supplementary Table 1). Patient and tumor characteristics for these three groups and respective *p*-values of comparative analysis are presented in Supplementary Table 2.

**Table 1:** Patient and tumor characteristics (*n* = 80).

**Characteristic**	**Data (%)**
**Gender**	
Male	58 (73)
Female	22 (27)
Median age	67 years
Range	21–89 years
**ECOG performance status**	
0	41 (51)
1	37 (46)
2	2 (3)
**Tumor site**	
Major SGAC	36 (45)
Parotid gland	33 (41)
Submandibular gland	1 (1)
Sublingual gland	0
Lacrimal gland	2 (3)
Minor SGAC	9 (11)
Nasal and paranasal sinus	5 (6)
Oral cavitiy	3 (4)
Oropharynx	1 (1)
SDAC	18 (23)
Parotid gland	14 (18)
Submandibular gland	2 (3)
Sublingual gland	1 (1)
Lacrimal gland	1 (1)
ITAC	17 (21)
paranasal sinuses	17 (21)
**Tumor Side**	
Unilateral	70 (88)
Bilateral	10 (12)
**Tumor classification**	
T2	17 (21)
T3	20 (25)
T4	43 (54)
**Node classification**	
N0	32 (40)
N+	48 (60)
**Metastasis classification**	
M0	80 (100)
M1	0
**Tumor differentiation**	
G1-2	28 (35)
G3	34 (43)
Gx	18 (23)
**Lymphovascular invasion**	
L0	49 (61)
L1	21 (26)
Lx	10 (13)
**Perineural invasion**	
Pn0	42 (53)
Pn1	28 (35)
Pnx	10 (13)
**Tumor status**	
Naive	73 (91)
Recurrence	7 (9)
**Operability**	
Yes	65 (81)
No	15 (19)
**Resection status**	
R0	11 (14)
R1	15 (19)
R2	29 (36)
Rx	10 (13)
**Macroscopic tumor**	
Yes	37 (46)
No	43 (54)

*SGAC, salivary gland adenocarcinoma; SDAC, salivary duct adenocarcinoma; ITAC, intestinal-type adenocarcinoma; ECOG, Eastern Cooperative Oncology Group*.

### Treatment Characteristics

For treatment planning, a CT scan (native and with contrast media) in head-first supine position with a slice thickness of 3 mm was performed and the patients were immobilized with thermoplastic head masks. A current MRI which was matched to the CT scan in irradiation position for tumor demarcation was used for target delineation via SyngoVia (VB20, 2017, Siemens, Erlangen, Germany). The gross tumor volume (GTV) was defined as the delineated primary tumor. CTV1, including the macroscopic tumor (GTV) or tumor bed for carbon ion boost, and CTV2, including CTV1 and typical local and regional pathways of tumor spread for the IMRT base plan, were outlined. The lymphatic drainage was involved into the CTV2 in the majority of patients for N+ (*n* = 48, 60%). Prophylactic RT of the neck was performed in all remaining patients (*n* = 15, 19%) except patients who received neck dissection before RT and were staged pN0 (16, 20%). Critical structures like optic chiasm, optical nerves, brain stem, spinal cord, and eyes were spared according to the QUANTEC data as low as possible (16, 17). Photon RT was performed with TomoTherapy® (Accuray, Sunnyvale, California) and carbon ion boost in active raster-scanning technique. All patients received bimodal RT with IMRT doses between 50 and 56 Gy in 2 Gy single dose fractions to the CTV2 and a carbon ion boost to the CTV1 with 18 Gy (RBE) to 24 Gy (RBE) in 3 Gy (RBE) single dose fractions. CTV2 received at least 90% and CTV1 received at least 95% of the prescription isodose. CIRT was applied in 5–6 fractions per week at the Heidelberg Ion Beam Therapy Center (HIT) with active raster-scanning and daily position correction. IMRT was applied in 5 fractions per week with a daily portal image guidance and weekly performed MV-CT scans for position correction. For improved comparability, the equivalent dose in 2 Gy per fraction was calculated using the formula EQD2 = D × ((d + α/β)/(2 + α/β)) (D = total dose in Gy; d = fraction dose in Gy; α/β = 2). Detailed treatment characteristics for all patients are shown in Table 2. Treatment characteristics for the three groups of adenocarcinoma (SGAC, SDAC, ITAC) and respective *p*-values of comparative analysis are presented in Supplementary Table 2.

**Table 2:** Treatment characteristics (n = 80).

**IMRT+C12**	**Data (%)**
**RT setting: postoperative/primary, n**	
Postoperative	65 (81)
Primary	15 (19)
**neck dissection**	
Yes	42 (52)
No	38 (48)
**RT of the neck**	
Yes	64 (80)
For pN+	26 (33)
For cN0 (prophylactic)	15 (19)
for cN+	23 (29)
No	16 (20)
Fractionation IMRT	25–28 x with 5 x/wk
Fractionation C12	6–8 x with 5–6 x/wk
**Treatment regimes**	
50 Gy/2 Gy IMRT+24 Gy/3 Gy (RBE) C12	47 (59)
52 Gy/2 Gy IMRT+18 Gy/3 Gy (RBE) C12	2 (3)
54 Gy/2 Gy IMRT+18 Gy/3 Gy (RBE) C12	14 (18)
56 Gy/2 Gy IMRT+18 Gy/3 Gy (RBE) C12	17 (21)
Median total dose in EQD2, range	80 Gy (RBE), 75–80 Gy (RBE)
Median CTV1 (C12), range	133, 25–353 ccm
Median CTV2 (IMRT), range	345, 47–980 ccm

*IMRT, intensity modulated radiotherapy; C12, carbon ions; RBE, relative biological effectiveness; EQD2, equivalent dose in 2 Gy fractions; CTV, clinical target volume*.

## Results

### Local Control and Survival Analysis

The median follow-up was 41 months (range, 9–130 months). At last follow-up, 25 patients (31%) had died of whom 19 patients (76%) had experienced a local, regional, and/or distant recurrence before. Overall, local recurrence was seen in 13 patients (16%), regional recurrence in 5 patients (6%), and distant recurrence in 23 patients (29%). The median time to local, regional and distant relapse after RT was 15 months (range, 4–66 months), 16 months (range, 4–26 months), and 12 months (range, 2–70 months), respectively. Best response with CR in 45 patients (56%), PR in 12 patients (15%) and SD in 23 patients (28%) could be achieved by bimodal RT.

**Figure 1:**
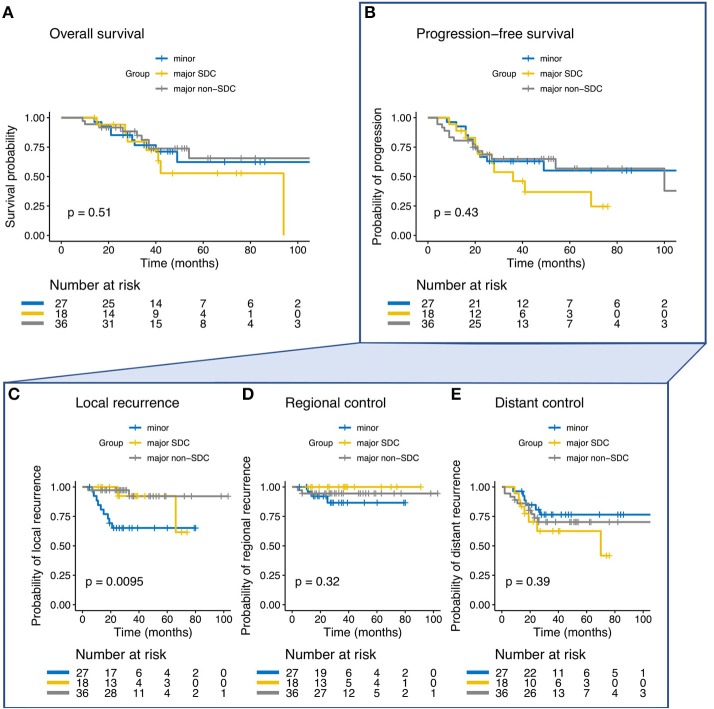
**(A–E)** Kaplan-Meier estimates and *p*-values for overall survival, progression-free survival (PFS), local control, regional control, and distant control in dependence of the three groups of salivary gland adenocarcinoma (SGAC), salivary duct adenocarcinoma (SDAC), and intestinal-type adenocarcinoma (ITAC).

The 3-year and estimated 5-year Kaplan-Meier rates for all patients were 83 and 75% for LC, 74 and 50% for OS and 60 and 53% for PFS, respectively. Bimodal RT for SGAC and SDAC resulted in a 3-year and estimated 5-year LC rate of 93% for both groups and RC rate of 94% for SGAC and 100% for SDAC, respectively (Figures 1C,D). In contrast, ITAC showed a decreased LC and RC compared to SGAC and SDAC (Figures 1C,D) with a 3-year LC rate of 50% (*p* < 0.01) and a 3-year RC rate of 76% (*p* = 0.069) according Kaplan-Meier estimates. 5-year LC and RC were not achieved by ITAC patients. Regarding OS and PFS, a 3-year OS of 88% for SGAC, 78% for SDAC and 67% for ITAC (Figure 1A; *p* = 0.08) and a 3-year PFS of 72% for SGAC, 53% for SDAC, and 44% for ITAC could be identified (Figure 1B; *p* = 0.063). Kaplan-Meier estimates for OS, PFS, LC, RC, and DC for the three groups are depicted in Figures 1A–E. Estimation of the correlation between the three endpoints local control (LC), overall survival (OS) and PFS is depicted in Supplementary Figures 1A,B.

Regarding primary vs. postoperative RT, univariate, and multivariate analysis showed no significant difference in OS (*p* = 0.15) and LC (*p* = 0.42) between the two subgroups. Nevertheless, PFS for postoperatively irradiated patients was superior in comparison with patients who were irradiated definitely (*p* < 0.01; Table 3, Supplementary Figure 2A, Supplementary Table 3).

**Table 3:** Multivariate analysis for LC, OS, and PFS.

**Variable**	**HR (95%-CI)**	***p*-value**
**Local control**		
SDAC vs. SGAC	4.221 (0.522–34.14)	0.177
ITAC vs. SGAC	11.03 (1.851–65.72)	**0.008**
Bilateral vs. unilateral	22.61 (4.199–121.8)	**0.001**
**Overall survival**		
SDAC vs. SGAC	1.784 (0.561–5.675)	0.327
ITAC vs. SGAC	3.697 (1.186–11.53)	**0.024**
ECOG1 vs. ECOG0	4.748 (0.760–29.64)	0.096
ECOG2 vs. ECOG0	2.825 (1.088–7.335)	**0.033**
Gender (female)	0.474 (0.128–1.761)	0.265
N+ vs. N0	3.100 (1.205–7.976)	**0.019**
**Progression-free survival**		
SDAC vs. SGAC	1.421 (0.530–3.806)	0.485
ITAC vs. SGAC	2.023 (0.761–5.379)	0.158
ECOG1 vs. ECOG0	1.677 (0.705–3.988)	0.243
ECOG2 vs. ECOG0	1.840 (0.321–10.53)	0.493
Gender (female)	0.408 (0.146–1.143)	**0.048**
T3 vs. T2	0.937 (0.181–4.850)	0.938
T4 vs. T2	2.039 (0.543–7.664)	0.291
N+ vs. N0	2.980 (1.318–6.741)	**0.009**
Operable vs. inoperable	0.341 (0.141–0.826)	**0.017**
CTV1	1.001 (0.996–1.006)	0.721

*HR, hazard ratio; CI, confidence interval; SGAC, salivary gland adenocarcinoma; SDAC, salivary duct adenocarcinoma; ITAC, intestinal-type adenocarcinoma; ECOG, Eastern Cooperative Oncology Group; CTV, clinical target volume; p-value, probability value*.

### Prognostic Factors

**Figure 2:**
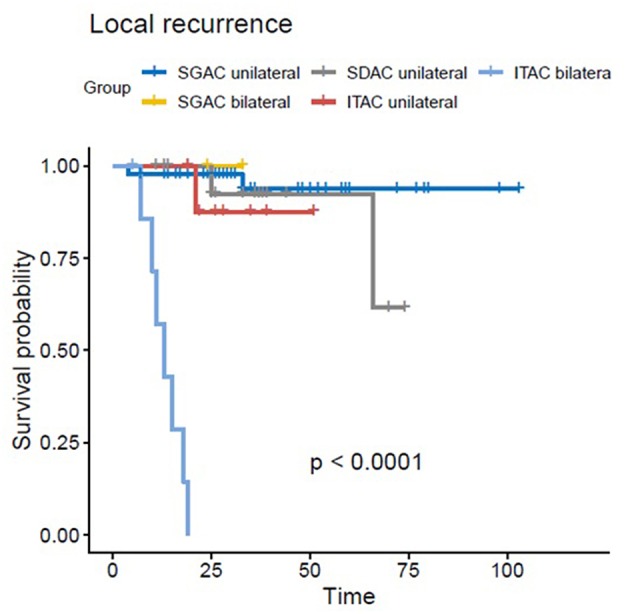
Kaplan-Meier estimates for bilateral vs. unilateral tumor side in dependence of the three groups of SGAC, SDAC, and ITAC with the worst LC for bilateral ITAC (*p* < 0.0001).

In the univariate and multivariate analysis, ITAC (vs. SGAC; *p* < 0.01, HR = 11.030, 95%-CI = 1.851–65.72) and bilateral tumor side (vs. unilateral; *p* < 0.01, HR = 22.612, 95%-CI = 4.199–121.78) were significantly associated with decreased LC. In Figure 2, Kaplan-Meier estimates for bilateral vs. unilateral tumor side are shown in dependence of the three groups of SGAC, SDAC, and ITAC (*p* < 0.0001). While a 3-year LC between 91 and 100% could be achieved for SGAC and SDAC, unilateral ITAC resulted in a LC of 87 vs. 0% for bilateral ITAC. All bilateral ITACs locally relapsed within 20 months post RT. Regarding OS, ITAC (vs. SGAC; Figure 1A; *p* = 0.0243, HR = 3.697, 95%-CI = 1.186–11.532), an ECOG performance score 2 (vs. ECOG 0; Supplementary Figure 2C; *p* = 0.0329, HR = 2.825, 95%-CI = 1.088–7.335) and N+ (vs. N0; Supplementary Figure 2D; *p* = 0.019, HR = 3.100, 95%-CI = 1.205–7.976) could be identified as independent negative prognostic factors. Additionally, female gender (vs. male; Supplementary Figure 2B; *p* = 0.0479, HR = 0.4079, 95%-CI = 0.146–1.143) and operable tumors (vs. inoperable; Supplementary Figure 2A; *p* = 0.0171, HR = 0.341, 95%-CI = 0.141–0.826) were associated with significantly increased and N+ (vs. N0; *p* = 0.009; HR = 2.980, 95%-CI = 1.318–6.741) with significantly worse PFS. Further statistical analyses demonstrated that all patients with a bilateral tumor were staged T4a (*n* = 3/10, 30%) or T4b (*n* = 7/10, 70%) with significant differences in the T stage compared to unilateral tumors (*p* < 0.001). Nevertheless, univariate and multivariate analyses did not show any significant impact of T stage on LC. In addition, bilateral tumors were associated with a higher CTV1 compared to unilateral tumors without significant relevance (Supplementary Figure 3, *p* = 0.36). Results of univariate analysis are shown in Supplementary Table 3. For results of multivariate analysis, please see Table 3.

### Acute and Late Toxicities

No acute grade ≥4 toxicities were observed. Acute grade 3 toxicities occurred in overall 17 patients (21%) while the most reported acute grade 3 toxicities were dysgeusia (*n* = 10, 12%), mucositis (*n* = 8, 10%), and dermatitis (*n* = 6, 7%). Regarding late grade ≥2 toxicities, the majority of patients claimed hearing impairment (*n* = 11, 14%), trismus (*n* = 12, 15%), dysosmia (*n* = 13, 16%), xerostomia (*n* = 21, 26%) and dysgeusia (*n* = 22, 27%). Overall, a total of 27 late grade ≥3 adverse side effects occurred in overall 20 patients (25%). Brain injury was diagnosed in 4 patients (5%) in median 19 months after RT (range, 14–36 moths) and was symptomatic in all 4 patients. Symptoms disappeared in 2 patients after oral cortisone treatment (grade 2, 2%) and surgery was necessary in the remaining 2 patients due to increasing symptoms under oral cortisone intake (grade 3, 2%). Osteoradionecrosis of the mandibular bone (*n* = 2, 2%) and the maxillary bone (*n* = 3, 4%) occurred in median 24 months (range, 8–45 months) after RT and were managed with conservative treatment methods, i.e., cortisone treatment, in 3 (grade 2, 4%) and surgery in 2 patients (grade 3, 2%). Acute hearing impairment was assessed in 6 patients (7%) and increased during follow-up. At last follow-up, 9 patients (11%) claimed hearing impairment, the majority was caused by chronic tympanic effusion (*n* = 7, 9%). Only one patient needed a hearing device due to severe hearing loss (1%). Visual impairment was relatively rare. One patient with a T4a paranasal adenocarcinoma of the maxillary sinus reported a moderate unilateral visual impairment (1%) while another patient with an advanced T4b tumor of the ethmoid sinus which infiltrated the skull base and the orbit developed unilateral visual loss 22 months after RT (grade 4, 1%). No further late grade 4 toxicity occurred. Acute and late adverse events grade ≥2 are shown in Table 4.

**Table 4:** Acute and late adverse events (grade ≥2).

	**Acute grade** **≥2, No. (%)**			**Late grade** **≥2, No. (%)**			
	**Grade 2**	**Grade 3**	**Total**	**Grade 2**	**Grade 3**	**Grade 4**	**Total**
Mucositis	23 (28)	8 (10)	31 (38)	0	0	0	0
Dermatitis	25 (31)	6 (7)	31 (38)	2 (2)	0	0	2 (2)
Xerostomia	14 (17)	1 (1)	15 (19)	17 (21)	4 (5)	0	21 (26)
Dysgeusia	27 (33)	10 (12)	37 (46)	18 (22)	4 (5)	0	22 (27)
Dysosmia	5 (6)	3 (4)	8 (10)	7 (9)	6 (7)	0	13 (16)
Trismus	7 (9)	0	7 (9)	10 (12)	2 (2)	0	12 (15)
Brain injury	0	0	0	2 (2)	2 (2)	0	4 (5)
Osteonecrosis	0	0	0	3 (4)	2 (2)	0	5 (6)
Mandibular bone	0	0	0	2 (2)	0	0	2 (2)
Maxillary bone	0	0	0	1 (1)	2 (2)	0	3 (4)
Cranial nerve dysfunction	2 (2)	0	2 (2)	5 (6)	2 (2)	0	7 (9)
Facial nerve	2 (2)	0	2 (2)	1 (1)	1 (1)	0	2 (2)
Trigeminal nerve	0	0	0	2 (2)	1 (1)	0	3 (4)
Recurrent nerve	0	0	0	2 (2)	0	0	2 (2)
Neuropathic pain	1 (1)	0	1 (1)	3 (4)	1 (1)	0	4 (5)
Visual impairment	2 (2)	0	2 (2)	1 (1)	1 (1)	1 (1)	3 (4)
Due to cataract	0	0	0	0	1 (1)	0	1 (1)
Hearing impairment	6 (7)	0	6 (7)	8 (10)	1 (1)	0	9 (11)
Due to tympanic effusion	6 (7)	0	6 (7)	6 (7)	1 (1)	0	7 (9)
Wound healing disorder	0	0	0	1 (1)	1 (1)	0	2 (2)

## Discussion

### Treatment

General treatment guidelines for adenocarcinomas of the head and neck are lacking. For each patient, treatment decision has to be individualized according age, ECOG status of the patient, tumor histology, tumor location and tumor stage. The National Comprehensive Cancer Network (NCCN) recommends surgical excision for all minor and major SGACs assuming operability. Nevertheless, in the majority of cases, RT is recommended due to the inoperability of the tumor, incomplete surgical margins (e.g., R1- or R2-resection), risk factors, i.e., undifferentiated or high-grade tumors, perineural invasion (PNI), lymphatic/vascular invasion (LVI), advanced tumor stages, or in case of rejection of the required operation (3). Although a negative patient selection is known for patients who require postoperative RT, several studies, mostly based on retrospective data, reveal the superiority of surgery plus postoperative RT compared to surgery alone for SGCs (14, 18). For adenocarcinomas of the ethmoid sinus, Choussy et al. showed in a retrospective multicenter study of 418 patients a significant survival advantage for patients treated with radical surgery and postoperative RT compared to patients who received RT alone (19). For major salivary glands of the head and neck, Mahmood et al. showed a significant survival benefit especially for patients with adenocarcinoma when treating them with adjuvant RT (20). In the current analysis, the majority of patients received postoperative RT (*n* = 66, 81%) and only 19% received RT alone due to inoperability of the tumor (*n* = 15). Nevertheless, R0 resection could be achieved in only 11 of 66 patients. Due to the radio resistance of adenocarcinoma, the high rate of residual tumor after surgery and the lacking evidence, we used to irradiate patients who received postoperative or primary radiotherapy with the same intensified doses. Since 2009, the Heidelberg Ion-Beam Treatment Center (HIT) routinely apply a combination treatment for MSG tumors with doses up to 80 Gy (relative biological effectiveness, RBE) using IMRT with 50–56 Gy and carbon ion boost with 18 Gy (RBE) to 24 Gy (RBE) attributed to previous phase I and II studies (21, 22). The carbon ion RBE was assumed to be 3 according to the biophysical Local Effect Model (LEM) (23). First experiences with bimodal RT including a combination regime of carbon ion boost with 18 Gy (RBE) in 3 Gy (RBE) single fractions in combination with IMRT with 54 Gy in 1.8 Gy single fractions applied for radio resistant adenoid cystic carcinoma of the head and neck were based on a phase-I/II study by the Society of Heavy Ion Research (GSI, Darmstadt, Germany) in cooperation with the Department of Radiation Oncology, University Hospital Heidelberg showing excellent treatment outcome for bimodal RT vs. photon beam RT (22). In this trail, Schulz-Ertner et al. showed superior 4-year LC control rates of 77.5 vs. 24.6% for bimodal RT vs. photon beam RT (21, 22). In the following years, Jensen et al. could show in the prospective COSMIC-trial more favorable treatment outcome with bimodal RT by escalating the applied carbon ion boost from 18 Gy (RBE) to 24 Gy (RBE) in 3 Gy (RBE) single fractions with a 3-year LC, PFS and OS of 81.9, 57.9, and 78.4% for MSGTs, comparable with Japanese data for carbon ion RT alone (24–26). As adenocarcinomas are known for their relative radio resistance, RT of a higher relative biological effectiveness (RBE) than photon RT, e.g., neutrons and carbon ions, is recommended for these tumors (22, 27, 28). Although a 5-year LC of even 93% for MSGCs could be achieved with neutrons, carbon ion RT resulted in lower high-grade toxicity, i.e., brain injury, compared with neutron data and is therefore preferentially used in head and neck tumors (29–33).

Although nodal metastases are rare and occur in 14–20% of all SGC patients, it is known that this rate can increase significantly in advanced and high-grade tumors (34, 35). Chen et al. showed for cN0 staged minor and major SGC patients, that elective neck irradiation resulted in a 10-year nodal failure rate of 0 vs. 26% for patients who did not receive elective neck irradiation (36). In contrast, the neck should be routinely treated in SDAC and ITAC patients due to the high probability of tumor spread into regional nodes (37, 38). Therefore, each patient in the current study received a treatment of the unilateral neck in case of unilateral tumors and of the bilateral neck in case of midline involving tumors either in form of a neck dissection (*n* = 42, 52%), a prophylactic neck RT (*n* = 15, 19%), or both neck dissection and RT of the neck for pN+ (*n* = 26, 32%).

### Findings

The current study analyzed treatment outcome of a relatively heterogeneous group of adenocarcinoma of the head and neck including various histological subgroups. The large cohort size enabled us to subdivide patients into three prognostic groups of SGAC, SDAC, and ITAC according to histology. For all patients, an excellent 3-year LC of 83%, OS of 74% and PFS of 60% was achieved with bimodal RT including raster-scanning carbon ion boost for dose-escalation. The most favorable prognosis regarding LC, OS, and PFS was observed in SGACs and the worst prognosis in ITACs. However, in multivariate analysis, significant differences between these two subgroups were identified for LC (*p* < 0.01) and OS (*p* = 0.0243) only, but not for PFS (*p* = 0.158). Treatment outcome did not differ between the subgroup of SDAC and SGAC (*p* = 0.177 for LC; *p* = 0.327 for OS; *p* = 0.485 for PFS). Additionally, bilateral tumor side resulted in a worse LC (*p* < 0.01) in multivariate analysis. An ECOG score of 2 (*p* = 0.033) and N+ (*p* = 0.019) were significantly associated with worse OS and male gender (*p* = 0.048), N+ (*p* = 0.009) and inoperability of the tumor (*p* = 0.017) were identified as independent negative prognostic factors for PFS, respectively. Treatment was tolerated-well. Only one patient claimed late grade 4 unilateral visual loss 22 months after RT. Acute and late grade 3 toxicities occurred in overall 21% (*n* = 17) and 25% (*n* = 20) of the patients.

### Limitations

The major limitations of the study are its retrospective design and the limited patient number in each histological subgroup (SGAC vs. SDAC vs. ITAC) which makes especially the comparability of postoperative and primary RT difficult. Therefore, in accordance to the retrospective multicenter studies by the Japan Carbon-Ion Radiation Oncology Study Group (J-CROS), treatment modality (postoperative vs. primary RT) was assessed as one prognostic factors of several prognostic factors in univariate and multivariate analyses and not considered separately as main point of the study (39). Due to the rareness of the disease and the variety of dose calculation models for carbon ion RT, sufficiently powered studies to make a clear comparison of the literature and determine the contribution of the addition of carbon ion RT to IMRT either in a primary or postoperative setting are lacking. In addition, the role of alternative fractionation schedules, i.e., accelerated RT, remains unclear due to missing data. In addition, although carbon ion RT seem to be a novel and beneficial treatment method especially for radio resistant tumors, the low availability of this treatment regime limits applicability of the used treatment method world-wide.

### Prognosis and Prognostic Factors

Data regarding prognosis and treatment outcome for several treatment regimes, i.e., surgery alone, surgery in combination with RT, RT alone, and different RT modalities, i.e., photon RT, neutron RT, heavy ion RT, are reported for patients with adenoid cystic carcinoma (ACC) and mucoepidermoid carcinoma (MEC) of the salivary glands by several authors (40–42). In contrast, SGACs are mostly considered as a subgroup among different other histologies of SGCs in several studies and data for SDAC and ITAC are limited to case reports and case series due to the rarity of these tumors. In addition, treatment results for adenocarcinoma treated with high-LET RT are rarely described in the literature which makes the interpretation of our results and the comparison with other studies difficult. While 5-year survival rates of 65% for overall SGC, 35–70% for ACC, 75–89% for low-grade MEC and 23–50% for high-grade MEC are described, 5-year survival for adenocarcinoma of the head and neck ranges between 20 and 100% in dependence of the histological subtype and tumor stage (3, 43). Polymorphic low-grade and basal cell adenocarcinoma show the best and salivary duct adenocarcinoma the worst prognosis with a 5-year survival of 95–100% vs. 20–50% among all adenocarcinomas of the salivary glands in the head and neck (3, 44).

In a large retrospective study of 565 SGC patients, Terhaard et al. could not identify significant differences in the LC (5-year LC of 87%) and OS (5-year OS of 64%) rate for adenocarcinoma of the head and neck compared with other histologies, i.e., ACC, acinic cell carcinoma or MEC, but in the distant-progression-free survival (DPFS; DPFS of 76% for all histologies vs. 67% for adenocarcinoma). Feinstein et al. reported a median OS of 5.5 years for SGAC and 4.7 years for SDAC patients and a median recurrence-free survival of 8.8 years for SGACs and 2.7 years for SDACs after surgery and postoperative RT (45). Age at diagnosis, tumor site (major vs. minor glands) and advanced nodal stage (N2) were identified as prognostic factors for OS and recurrence-free survival. For non-ACC SGCs (eight patients with SGAC) treated with IMRT plus carbon ion boost, Jensen et al. showed a superior 5-year LC for SGACs and salivary gland acinic cell carcinomas of 100 vs. 67% for MEC. Nevertheless, SGACs had the least favorable outcome in the PFS and DC (46). In a retrospective multicenter study, Saitoh et al. reported a 5-year OS and LC of 60 and 79% for 47 adenocarcinomas of the head and neck with differing sites of origin who were treated by carbon ions only with a median total dose of 64 Gy (RBE) at 4 Gy (RBE) single fractions either in a primary (*n* = 28) or postoperative (*n* = 19) setting (39). Operability of the tumor and hypo fractionation were identified as independent prognostic factors for OS.

For SDACs, a 5-year LC rate of 58% and a DPFS rate of 57% were shown by Di et al. gross residual tumors after surgery resulting in a significantly decreased LC and DPFS (47). Gilbert et al. reported a 5-year OS of 40% and a 5-year disease-free survival of 26% for SDAC patients who received surgery and adjuvant radiotherapy in the majority of cases (12). Higher age, advanced T and N stage, extra capsular spread, perineural invasion, and facial nerve sacrifice were associated with worse prognosis (12, 48).

For ITACS, prognosis is poor as well, as 5-year survival rates of 50% for ITACs associated to wood dust exposure and 20–49% for sporadic ITACs are reported (49). Deaths are preferentially caused by local relapses which occur with a 5-year probability of 51–59% after surgery alone and 23% (carbon ion RT) to 38% (photon beam RT) after surgery and postoperative RT (6, 50). In general, a mean local recurrence rate of 30% is described in the literature (a27) while 80% of the relapses occur within the first 3 years after treatment (37). TNM stage with an OS of 80% for T1 and 25% for T4 stage, tumor differentiation, skull base invasion, and resection margins are mostly discussed as prognostic factors (13).

### Toxicity

More conformal RT techniques, i.e., IMRT, the development of high linear-energy transfer RT, i.e., neutron RT or carbon ion RT, and improved imaging procedures, i.e., positron emission tomography (PET-CT), have led to decreased toxicity rates compared with conventional or 3-dimensional RT in head and neck cancer (27, 28). Nevertheless, certain late adverse effects, i.e., brain injury, osteoradionecrosis, hearing impairment/hearing loss, visual impairment/visual loss, nerve palsy and fistula, are still diagnosed to a large extent after RT and can occur years after the completion of the treatment (39, 44, 46, 51, 52). Thus, Mendenhall et al. reported severe late side effects in overall 13 of 224 SGC patients (6%), consisting of osteoradionecrosis (*n* = 4, 2%), unilateral vision loss (*n* = 6, 3%), fistula (*n* = 1, 0.4%), submental abscess (*n* = 1, 0.4%), and secondary malignancy (*n* = 1, 0.4%) who were treated with definite or postoperative photon beam RT (51). In accordance to Mendenhall's results, Holtzman et al. showed osteoradionecrosis in 5% (*n* = 14/291) and vision/hearing loss in 6% (*n* = 17/291) of their patients treated with the same method (52). For bimodal RT including IMRT and CIRT, Jensen et al. could identify hearing impairment in 13% (*n* = 5/40) of the patients with non-ACC SGCs (46). At a relatively short median follow-up of 26 months, hearing loss, visual loss, brain injury and osteoradionecrosis did not appear. In a further study which included 6 patients (2 patients received postoperative RT and 4 patients received primary RT) with basal cell adenocarcinoma treated with carbon ions only (total dose of 64 Gy (RBE) in 4 Gy (RBE) single dose fractions), Jingu et al. observed one grade 4 visual loss 12 months and one facial nerve palsy 6 months after treatment (44). Nevertheless, several authors described a median latency of 5–8 years (range <1–20 years) for these symptoms (46, 52). Especially regarding visual loss, Saitoh et al. observed grade 3 and 4 visual impairment in 9% (*n* = 4/47) and 11% (*n* = 5/47) of the patients with an occurrence of blindness in median 31 months (range 19–62 months) after carbon ion RT (39). Further severe late adverse effects were osteoradionecrosis in 11% (*n* = 5/47), cranial nerve palsy in 6% (*n* = 3/47), and brain injury in 6% (*n* = 3/47) of the cases. Several studies described a dose-volume-dependence between the maximum doses delivered to a certain volume of the organ at risk and the occurrence of symptoms (53–55). In the current study, comparable late adverse side effects (≥ grade 2) were observed for bimodal RT with visual impairment in 4% (*n* = 3/80), visual loss in 1% (*n* = 1/80), brain injury in 5% (*n* = 4/80), osteoradionecrosis in 6% (*n* = 5/80), and cranial nerve palsy in 9% (*n* = 7/80) of our patients. Nevertheless, considerably less late grade 4 side effects occurred with only one case of total unilateral blindness (1%).

## Conclusion

Radiotherapy including active raster-scanning carbon ion boost for relatively radio resistant adenocarcinomas of the head and neck resulted in favorable local control and survival outcome with moderate toxicity for salivary gland and salivary duct adenocarcinomas compared to photon data described in the literature. However, local control and prognosis for bilateral ITAC s seem to remain low even after dose-escalation.

## Data Availability

All data generated or analyzed during the current study are included in this published article. The dataset is available from the corresponding author on reasonable request.

## Ethics Statement

The final protocol was approved by the ethics committee of the University of Heidelberg, Germany (S-421/2015).

## Author Contributions

SAk, SAd, and JD planned and supervised this analysis as part of the head and neck research group. SAk performed data collection and review. AM performed all statistical analysis. SAk reviewed data analysis and drafted the manuscript. SAk, AM, JH-R, TH, SK, TF, CF, SR, KH, PP, JD, and SAd contributed patient data and participated in reviewing and improving analysis and manuscript. All authors read and approved the final manuscript.

### Conflict of Interest Statement

The authors declare that the research was conducted in the absence of any commercial or financial relationships that could be construed as a potential conflict of interest.
