# Supplementary material for: Treatment Outcome of a Combined Dose-Escalated Treatment Regime With Helical TomoTherapy® and Active Raster-Scanning Carbon Ion Boost for Adenocarcinomas of the Head and Neck
Source: Front Oncol. 2019 Aug 13;9:755. doi: 10.3389/fonc.2019.00755 (PMC6705231; doi:10.3389/fonc.2019.00755)
Supplement: Supplementary file 1 [file Data_Sheet_1.docx]

**Suppl. Tables:**

| **Suppl. table 1.** Histological subtypes of all tumors | |
| --- | --- |
| *Characteristic* | *Data* |
| No. of patients | 80 |
| SGAC | 45 |
| NOS | 35 |
| basal cell | 7 |
| polymorphous | 2 |
| oncocytic | 1 |
| SDAC | 18 |
| ITAC | 17 |
| *Abbreviations:* SGAC= salivary gland adenocarcinoma, SDAC= salivary duct adenocarcinoma, ITAC=intestinal-type adenocarcinoma, NOS=not otherwise specified | |

| **Suppl. table 2.** Patient, tumor, treatment characteristics and p-values of comparative analysis between 3 groups (minor SGC, major SGC and major SDC are presented) | | | | | |
| --- | --- | --- | --- | --- | --- |
| *Characteristic* | *Total (n=80)* | *SGAC (n=45)* | *SDAC (n=18)* | *ITAC (n=17)* | *p-value* |
| Gender: male/female, n | 58/22 | 27/18 | .16/2 | .15/2 | **0.020** |
| Median Age, range (y) | 67, 21-89 | 67, 11-88 | 69, 41-84 | 67, 20-81 | 0.271 |
| ECOG performance status: 0/1/2, n | 41/37/2 | .22/23/0 | .9/7/2 | .10/7/0 | 0.681 |
| Tumor side: unilateral/bilateral | 70/10 | .43/2 | .18/0 | .9/8 | * |
| Tumor classification: T2/T3/T4, n | 17/20/43 | .13/13/19 | .1/7/10 | .3/0/14 | **0.005** |
| Node classification: N0/N+, n | 32/48 | .19/26 | .10/8 | .3/14 | 0.064 |
| Metastasis classification: M0/M1, n | 80/0 | .45/0 | .18/0 | .17/0 | N/A |
| Tumor differentiation: G1-G2/G3/unclassified**, n | 28/34/18 | .14/19/12 | .3/10/5 | .11/5/1 | 0.054 |
| Lymphovascular invasion: LV0/LV1/unclassified**, n | 49/21/10 | .29/11/5 | .10/5/3 | .10/5/2 | 1 |
| Perineural invasion: Pn0/Pn1/unclassified**, n | 42/28/10 | .25/15/5 | .4/11/3 | .13/2/2 | **0.004** |
| Tumor status: naive/recurrence, n | 73/7 | .41/4 | .18/0 | .14/3 | 0.130 |
| Operability: yes/no, n | 65/15 | .35/10 | .15/3 | .15/2 | 0.740 |
| Resection status: R0/R1/R2/unclassified**, n | 11/15/29/10 | .6/7/17/5 | .1/8/3/3 | .4/0/9/2 | **0.005** |
| Macroscopic tumor: yes/no, n | .44/36 | .27/18 | .6/12 | .11/6 | 0.119 |
| Median CTV1 (C12), range (ccm) | 133 , 25-353 | 133, 25-323 | 122, 36-259 | 133, 57-240 | 0.813 |
| Median CTV2 (IMRT), range (ccm) | 345 , 47-980 | 344, 47-765 | 344, 105-742 | 344, 154-980 | 0.825 |
| *Abbreviations:* SGAC= salivary gland adenocarcinoma, SDAC= salivary duct adenocarcinoma, ITAC=intestinal-type adenocarcinoma *not assessable in order to missing bilateral data for SDAC and ITAC, ECOG=Eastern Cooperative Oncology Group. **unclassified samples were not included in the enrichment analysis. | | | | | |

| **Suppl. table 3.** Univariate analysis for LC, OS and PFS | | | | | | |
| --- | --- | --- | --- | --- | --- | --- |
|  | **LC** | | **OS** | | **PFS** | |
| *Variable* | *HR (95%-CI)* | *p-value* | *HR (95%-CI)* | *p-value* | *HR (95%-CI)* | *p-value* |
| SDAC vs. SGAC | 2.532 (0.356-17.99) | 0.353 | 2.366 (0.877-6.384) | 0.089 | 2.214 (0.975-5.029) | 0.058 |
| ITAC vs. SGAC | 19.59 (3.760-102.1) | **<0.001** | 2.643 (1.001-6.980) | **0.049** | 2.276 (1.002-5.169) | **0.049** |
| Age (+10 years) | 0.885 (0.633-1.237) | 0.473 | 1.215 (0.880-1.678) | 0.237 | 1.169 (0.906-1.509) | 0.230 |
| ECOG1 vs. ECOG0 | 1.632 (0.517-5.156) | 0.404 | 2.285 (0.929-5.624) | 0.072 | 2.317 (1.111-4.832) | **0.025** |
| ECOG2 vs. ECOG0 | * | * | 7.676 (1.565-37.65) | **0.012** | 3.526 (0.774-16.07) | 0.103 |
| Gender (female) | 0.424 (0.092-1.953) | 0.257 | 0.293 (0.087-0.985) | **0.035** | 0.409 (0.169-0.990) | **0.041** |
| T3 vs. T2 | * | * | 1.120 (0.186-6.755) | 0.902 | 1.010 (0.2234-4.57) | 0.989 |
| T4 vs. T2 | * | * | 3.231 (0.747-13.98) | 0.117 | 3.765 (1.139-12.45) | **0.030** |
| N+ vs. N0 | 0.590 (0.158-2.208) | 0.434 | 2.566 (1.102-5.976) | **0.024** | 2.144 (1.073-4.283) | **0.027** |
| G3 vs. G1/G2 | 0.425 (0.106-1.700) | 0.212 | 1.678 (0.650-4.339) | 0.280 | 1.257 (0.570-2.773) | 0.570 |
| LV1 vs. LV0 | 0.472 (0.102-2.186) | 0.326 | 1.297 (0.509-3.309) | 0.585 | 1.175 (0.542-2.550) | 0.683 |
| Pn1 vs. Pn0 | 0.399 (0.083-1.925) | 0.236 | 0.764 (0.292-1.999) | 0.583 | 1.140 (0.540-2.404) | 0.731 |
| Bilateral vs. unilateral | 27.79 (7.653-100.9) | **<0.001** | 2.566 (0.986-6.679) | 0.053 | 2.253 (0.975-5.208) | 0.057 |
| Recurrent vs. primary | 1.227 (0.157-9.593) | 0.845 | 0.489 (0.111-2.152) | 0.335 | 0.425 (0.101-1.800) | 0.231 |
| Operable vs. inoperable | 0.586 (0.155-2.210) | 0.424 | 0.531 (0.224-1.255) | 0.143 | 0.362 (0.178-0.736) | **0.004** |
| R1 vs. R0 | * | * | 3.500 (0.391-31.37) | 0.263 | 1.513 (0.361-6.347) | 0.572 |
| R2 vs. R0 | * | * | 1.884 (0.227-15.65) | 0.558 | 1.024 (0.277-3.790) | 0.972 |
| CTV1 | 1.006 (0.999-1.013) | 0.070 | 1.004 (0.999-1.009) | 0.167 | 1.004 (1.000-1.009) | **0.036** |
| *Abbreviations*: LC= local control, OS=overall survival, PFS=progression-free survival, HR=hazard ratio, CI=confidence interval, SGAC= salivary gland adenocarcinoma, SDAC=salivary duct adenocarcinoma, ITAC=intestinal-type adenocarcinoma, ECOG=Eastern Cooperative Oncology Group, CTV=clinical target volume. * Could not be reliably calculated due to lack of events | | | | | | |
