# Supplementary material for: Treatment Outcome of a Combined Dose-Escalated Treatment Regime With Helical TomoTherapy® and Active Raster-Scanning Carbon Ion Boost for Adenocarcinomas of the Head and Neck
Source: Front Oncol. 2019 Aug 13;9:755. doi: 10.3389/fonc.2019.00755 (PMC6705231; doi:10.3389/fonc.2019.00755)
Supplement: Supplementary file 2 [file Data_Sheet_2.docx]

Suppl. Figures

**
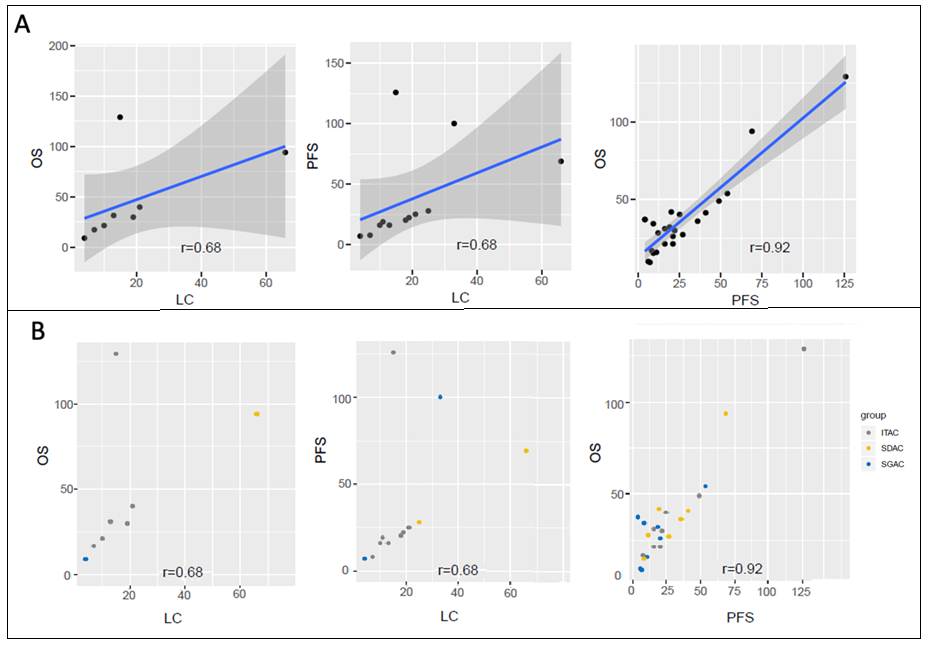
**

**Suppl. Figure 1A-B.** Estimation of the correlation between the three endpoints local control (LC), overall survival (OS) and progression-free survival (PFS). Of note, the data show only cases, in which both endpoints occurred. (A) shows a correlation plot between respective pairs of endpoints. Exemplarily, linear regression analysis showed that OS was more explained by PFS (r=0.92; adjusted R^2^=0.842), than by LC (r=0.68; adjusted R^2^=0.168). The impact of LC on PFS was similar to the impact on OS (r=0.68; adjusted R^2^=0.128). (B) shows the same visualization stratified for the three groups of adenocarcinomas.


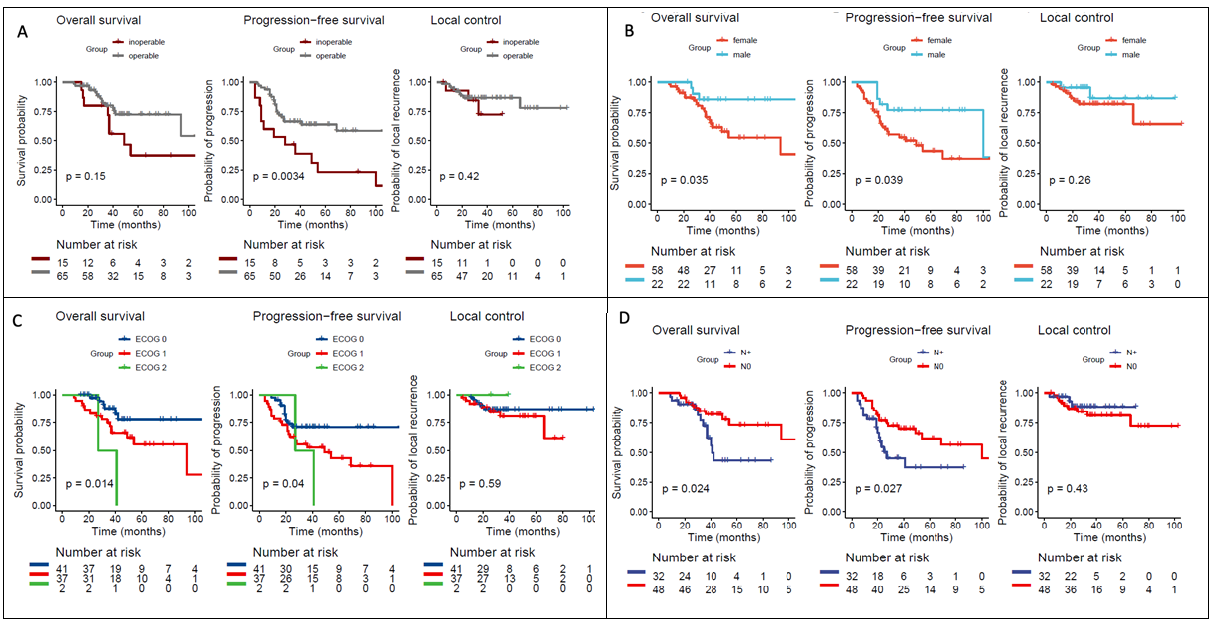


**Suppl. figure 2A-D.** Kaplan-Meier estimates and p-values for LC, OS and PFS in dependence of operability vs. inoperability (A), female vs. male gender (B), ECOG score (C) and N+ vs. N0 (D). In multivariate analysis, ECOG performance score 2 (p=0.0329) and N+ (p=0.019) could be identified as independent negative prognostic factors for OS. Female gender (p=0.0479) and operable tumors (p=0.0171) were associated with significantly increased and N+ (p=0.009) with significantly worse PFS.


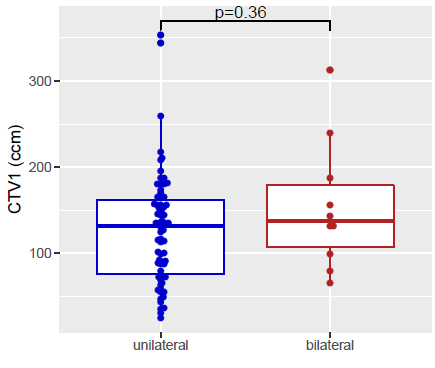


**Suppl. figure 3.** Box plot comparing the distribution of the CTV1 between the two groups of bilateral and unilateral tumors. No significant correlation was seen between tumor side and CTV1 although bilateral tumors were associated with a higher CTV1 compared to unilateral tumors (p=0.36).
